# Identification of QTLs/ Candidate Genes for Seed Mineral Contents in Common Bean (*Phaseolus vulgaris* L.) Through Genotyping-by-Sequencing

**DOI:** 10.3389/fgene.2022.750814

**Published:** 2022-03-14

**Authors:** Muslima Nazir, Reetika Mahajan, Sheikh Mansoor, Sheezan Rasool, Rakeeb Ahmad Mir, Ravinder Singh, Vandana Thakral, Virender Kumar, Parvaze A. Sofi, Hamed A. El-Serehy, Daniel Ingo Hefft, Sajad Majeed Zargar

**Affiliations:** ^1^ Proteomics Laboratory, Division of Plant Biotechnology, Sher-e-Kashmir University of Agricultural Sciences and Technology of Kashmir, Srinagar, India; ^2^ Division of Biochemistry, FBSc, Sher-e Kashmir University of Agricultural Sciences and Technology of Jammu, Jammu, India; ^3^ Department of Biotechnology, BGSB University, Rajouri, India; ^4^ School of Biotechnology, Sher-e-Kashmir University of Agricultural Sciences and Technology of Jammu, Jammu, India; ^5^ National Agr Food Biotechnology Institute (NABI), Mohali, India; ^6^ Division of Genetics and Plant Breeding, Sher-e-Kashmir University of Agricultural Sciences and Technology of Kashmir, Srinagar, India; ^7^ Department of Zoology, College of Science, King Saud University, Riyadh, Saudi Arabia; ^8^ University Centre Reaseheath, Reaseheath College, Nantwich, United Kingdom

**Keywords:** common bean, single nucleotide polymorphism (SNP), genome wide association studies (GWAS), ionome, transporters, population structure

## Abstract

Throughout the ages, the common bean has been consumed by humanity as an important food staple crop and source of nutrition on a global scale. Since its domestication, a wide spectrum of phenotypic and genotypic investigations have been carried out to unravel the potential of this crop and to understand the process of nutrient accumulation along with other desirable characteristics. The common bean is one of the essential legume crops due to its high protein and micronutrient content. The balance in micronutrients is critical for the growth and development of plants as well as humans. Iron (Fe), Zinc (Zn), Copper (Cu), Manganese (Mn), Magnesium (Mg), Calcium (Ca), and Molybdenum (Mo) are some of the important micronutrients present in legumes. Thus, we aimed to investigate the quantitative trait loci’s (QTLs)/single nucleotide polymorphisms (SNPs) to identify the candidate genes associated with micronutrients through genotyping by sequencing (GBS). In our investigation, through GBS we identified SNPs linked with traits and assessed seven micronutrients in 96 selected common bean genotypes for screening nutritionally rich genotypes. Among 96399 SNPs total identified through GBS, 113 SNPs showed significant phenotypic variance, ranging from 13.50 to 21.74%. SNPs associated with most of the seed micronutrients (Mg, Mn, Fe, Ca, Cu) were found on chr3 & chr11 (Mg, Mn, Mo, Ca, Zn). The findings from this study could be used for haplotype-based selection of nutritionally rich genotypes and for marker-assisted genetic enhancement of the common bean. Further, the identified SNPs for candidate genes/transporters associated with micronutrient content may pave the way for the enrichment of seeds by employing genomics-assisted breeding programs.

## 1 Introduction

Common bean (*Phaseolus vulgaris* L.), an important food legume, constitutes 50% of the grain legumes consumed as a human food source (Câmara et al., 2013; FAOSTAT, 2017). Common bean has huge genetic variation and based on domestication it is distinguished into two main gene pools, Andean and Mesoamerican. Cultivars in the mesoamerican gene pool have small to medium seed size and with “S” or “B” phaseolin patterns, while Andean cultivars have a large seed size with phaseolin patterns “T”/“C”/“H”/“A” (Bitocchi et al., 2012; Bellucci et al., 2014).

Being a great source of carbohydrates, dietary proteins, soluble and insoluble fibers, vitamins, and essential micronutrients such as minerals including Iron (Fe), Zinc (Zn), Copper (Cu), Manganese (Mn), Magnesium (Mg), Calcium (Ca) and Molybdenum (Mo), beans have often been considered a “poor man’s meat” (Fennema owen, 2000; Hayat et al., 2014; Yeken, 2018). These micronutrients play a pivotal role in the proper growth and development of plants and animals. The deficiency of any essential micronutrients hinders the proper functioning of biological processes leading to several metabolic and physiological implications. In many low- and lower-middle income countries, especially those in Asia, Africa, and Latin America, micronutrients Fe and Zn are the main components of hidden hunger (Darnton-Hill et al., 2006). Similarly, Fe and Zn deficiency cause severe yield loss in crops and metabolic disturbances in humans (Zargar et al., 2015). In addition, Cu is one of the important trace elements that play a vital role in maintaining metabolic activities. In humans, Cu deficiency leads to anemia, cardiac dysfunction, myeloneuropathy, and myelopathy, whereas in plants, its deficiency leads to lignification dysfunctioning (Geir, 2013; Lehmann and Rillig, 2015; Papamargaritis et al., 2015).

Food crops are the major source of essential minerals (Hardiman et al., 1984; Ali et al., 2014). Biofortification of nutrient-rich plants is important in addressing malnutrition-related issues. Thus, given today’s population explosion and food shortage issues, there is a need to introduce smart food crops into our diet. So far, research has been carried out using both conventional and modern breeding approaches to increase the nutrient content in cultivated plants. It is now important to identify genetic loci that regulate the uptake of essential minerals, as each genetic loci is an essential factor in the success of the biofortification breeding effort. Association mapping is one of the modern breeding approaches used to identify genetic loci that determine desired traits (Lewontin and Kojima, 1960). Association mapping has several advantages over the bi-parental QTL mapping approach (Yu and Buckler, 2006; Tian et al., 2011; Wen et al., 2014; Nadeem et al., 2021). For example, association mapping explores the allelic diversity that exists in the diverse germplasm, while QTL mapping can examine the allele variation present in only two parental lines. Undoubtedly, both approaches are indispensable and have their own advantages and disadvantages (Sonah et al., 2013). We believe an integrated approach involving both association mapping as well as biparental mapping can lead to a breakthrough in crop improvement.

Since the whole genome sequence of the common bean is available in the public domain, as such there is an excellent opportunity to perform Genome-wide association studies (GWAS) to identify QTLs followed by candidate genes that govern the uptake and accumulation of minerals (Schmutz et al., 2014). Genome-wide association study is one of the modern breeding approaches for mapping genes associated with different traits (Lu et al., 2015; Liu et al., 2016). The introduction of Next-generation sequencing (NGS) technologies has sped up the identification of SNPs and subsequent genotyping (Verma et al., 2015). Genotyping-by-sequencing (GBS) is a robust and cost-effective method wherein selective small genome fragments obtained by restriction digestion are sequenced by NGS platform to identify SNPs (Narum et al., 2013; Peterson et al., 2014; Schilling et al., 2014). In the last two decades, numerous studies have been performed using the GBS approach in diverse plant species, including wheat, canola, barley, and soybean, which are known to have a complex genome. In this regard, GWAS in common bean will allow us to estimate population structure and linkage disequilibrium (LD), connecting the variation in the genome with the phenotypic variations in the population. The LD-based analysis is organized based on population structure and genetic relatedness among populations. Several association studies with respect to micronutrient contents have been carried out in food crops like rice (Shao et al., 2011; Norton et al., 2014), pea (Diapari et al., 2015), chickpea (Diapari et al., 2014; Upadhyaya et al., 2016; Ozkuru et al., 2019), common bean (Nemli et al., 2016; Mahajan et al., 2017a; Erdogmus et al., 2020).

In the present study, we examined the germplasm of common beans of the Jammu and Kashmir region of northwestern Himalayas, India, for various micronutrients, followed by the identification of QTLs associated with the accumulation of nutrients. To identify genes/QTLs that regulate micronutrients in beans, GBS-based SNPs were discovered from natural populations of beans. Based on the preliminary studies, a set of 96 different bean genotypes was created, which were collected from other regions of the northwestern Himalayas (Zargar et al., 2014). In the present study, the ionome profile, which contains Ca, Cu, Mg, Mn, and Mo, of the bean core set was deciphered, and the QTLs contributing to their accumulation by GWAS have been identified. The investigation led us to inventory the micronutrients of the 96 different types of beans to determine candidate lines (nutrient-rich for different micronutrient levels in the seed) that can be used in breeding high-nutrient, high yielding bean varieties. In addition, the identification of QTLs can serve as critical genomic resources for improving the micronutrient profile in beans. These studies can improve the understanding of possible correlations for the accumulation of different elements. To understand the function of the respective candidate genes that regulate micronutrient uptake in beans, further studies need to be performed that may include knockout or overexpression of responsive candidate genes.

## 2 Experimental Procedures

### 2.1 Plant Material

A total of 96 common bean germplasm lines, mostly landraces and a few released varieties (SFB1, SR1, SR2, Arka Anoop, VLR-125) were used as plant material in the current study. Germplasm was collected from different geographical regions of Jammu and Kashmir (Supplementary Table S1) and maintained at the research fields at Division of Genetics and Plant Breeding, Faculty of Agriculture, Wadura, Sher-e-Kashmir University of Agricultural Sciences and Technology-Kashmir (SKUAST-K), Sopore. Most of the released varieties used in this study were developed through a single plant selection and have been used as checks.

### 2.2 Field Experiment and Micronutrient Profiling

Field experiments were conducted during 2016 and 2017 at the experimental field of the College of Agriculture, Wadura, SKUAST-Kashmir, India (34.34 North; 74.4 East; Altitude: 1,590.00 m). Clay loam textured soil with pH (7.2), organic carbon (.65%), electrical conductivity (.18 dS/m), CEC (16 meq/kg), and an available concentration of different elements in the soil i.e. P (4.91 mg kg^−1^), K (5.55 mg kg^−1^), Zn (.68 mg kg^−1^), Fe (5.1 mg kg^−1^), Cu (.29 mg kg^−1^), Mn (6.2 mg kg^−1^) was used for plantation of germplasm seeds in the experimental sites. The experiment was laid out according to augmented block design, which includes more than one released variety that is taken as replicated treatments, and these varieties are repeated in each block. Five released varieties (SFB1, SR1, SR2, Arka Anoop, VLR-125) were included in each block as checks. All the standard agronomical practices recommended were followed to raise healthy and disease-free crop plants. Harvesting was done at the time of 90% pod maturity. Further, the seed material of each genotype was powdered to analyze seven essential micronutrients i.e. Cu, Mn, Mg, Ca, Fe, Zn, and Mo. The elemental profiling of these genotypes was determined using a portable X-ray fluorescence spectrometer (pXFR). The pXRF instrument was calibrated as explained in Reidinger et al. (2012). In pXRF, a synthetic methylcellulose matrix was used to spike the known quantity of standard elements. Based on the methylcellulose pellet with know standards, an elemental composition standard curve was developed and subsequently used for sample evaluation. Similarly, samples were cross-verified with Energy Dispersive X-ray Fluorescence (ED-XRF).

### 2.3 Genotyping-by-Sequencing of Common Bean Genotypes

Genomic DNA was extracted from 15 day old leaves by using the CTAB method (Doyle and Doyle, 1990), and the quality, as well as quantity of DNA, was checked on both gel electrophoresis (.8% Agarose) and nano-drop (mySPEC, Wilmington, USA). The extracted genomic DNA was purified for the preparation of multiplex GBS libraries *via* Illumina HiSeq 2000 (SciGenom Pvt. Ltd., India). *Ape*K1 (from *Aeropyrum pernix K1*) restriction enzyme was employed for restriction digestion of genomic DNA. After quality filtering, de-multiplexed high-quality sequences were mapped to the reference common bean genome (Phtyozome v12.1 database), and SNPs were mined from the coding and non- coding regions of common bean genes and chromosomes. Subsequently, SNPs mined were structurally annotated on the diverse coding DNA sequence (CDS) and non-coding (upstream/downstream regulatory regions and introns) sequence components of genes and intergenic regions of the common bean genome.

### 2.4 Statistical Analysis

#### 2.4.1 Micronutrient Profiling Analysis

All the observations were recorded in replicates of three, and values were then averaged. One-way ANOVA was applied to evaluate the variance of seven micronutrients among the genotype and Pearson’s pairwise correlation coefficient was calculated for all elements using the SPSS program (ver. 16).

#### 2.4.2 Population Genetic Analysis

Population structure was estimated using a Bayesian Markov Chain Monte Carlo model (MCMC) implemented in STRUCTURE (Pritchard et al., 2000). Five runs were performed for each number of populations (K) set from 01 to 12. Burn-in time and MCMC replication number were both set to 100,000 for each run. The most likely *K value* was determined by the log probability of the data LnP(D) and delta K, based on the rate of change in LnP(D) between successive K values. These analyses were performed using Structure Harvester (Earl and vonHoldt, 2012). The neighbor-joining tree was built using Phylip and MEGA5 (Felsenstein, 1989; Tamura et al., 2011).

#### 2.4.3 Genome-wide Association Analysis

All the analyses were performed using TASSEL3.0 and the Genomic Association and Prediction Integrated Tool (GAPIT) (Bradbury et al., 2007; Lipka et al., 2012). Mixed linear models (MLM) were used for the identification of SNPs associated with these traits. Van Raden method (K) was used to calculate the kinship matrix (Loiselle et al., 1995; Hyun et al., 2008). Covariate like P from principal component analysis and Q from STRUCTURE along with kinship matrix (K) were used for mixed linear models (MLM). The negative log(1/n) was used to establish a significance threshold (Wang et al., 2012; Yang et al., 2013).

#### 2.4.4 LD Plots and Haplotype Blocks

The SNP matrix for all the samples was converted to HapMap format and TASSEL was used for the filtering out of SNP with major allele frequency (MAF) of less than .05. The LD plots for individual chromosomes were created in Tassel > Analysis > Diversity > Linkage Disequilibrium with LD Type as “Sliding Window” and LD Window size set to 50. The heterozygous sites were treated as missing and the R^2^ accumulated was calculated for 100 intervals with a size of .01. The R^2^ data obtained following LD analysis was used for plotting chromosome-wise LD information using MS Excel.

The PLINK v1.90b6.24 64-bit was used to determine the block size of SNP haplotypes. The individual chromosome Tassel files were saved to Plink format (.map and .ped) using the option available in Tassel. The haplotype blocks were calculated using—blocks no-pheno-req-- blocks-max-kb 100--make-founders command in Plink. The Haplotype Block analysis revealed the size of Haplo Blocks, including the number of SNPs on each block. The data was plotted using MS Excel.

### 2.5 Candidate Gene Identification

For candidate genes identification, the reference genome of *P. vulgaris* (V2.1) was used. The candidate genes were identified in .1 Mb both flanking regions of significant SNPs using the BioMart tool (Smedley et al., 2009), and the information related to gene function, Pfam ID, Panther ID, KOG ID, gene ontology ID, and their description were downloaded.

## 3 Results

### 3.1 Genetic Variation in Common Bean Seed Micronutrients

**FIGURE 1:**
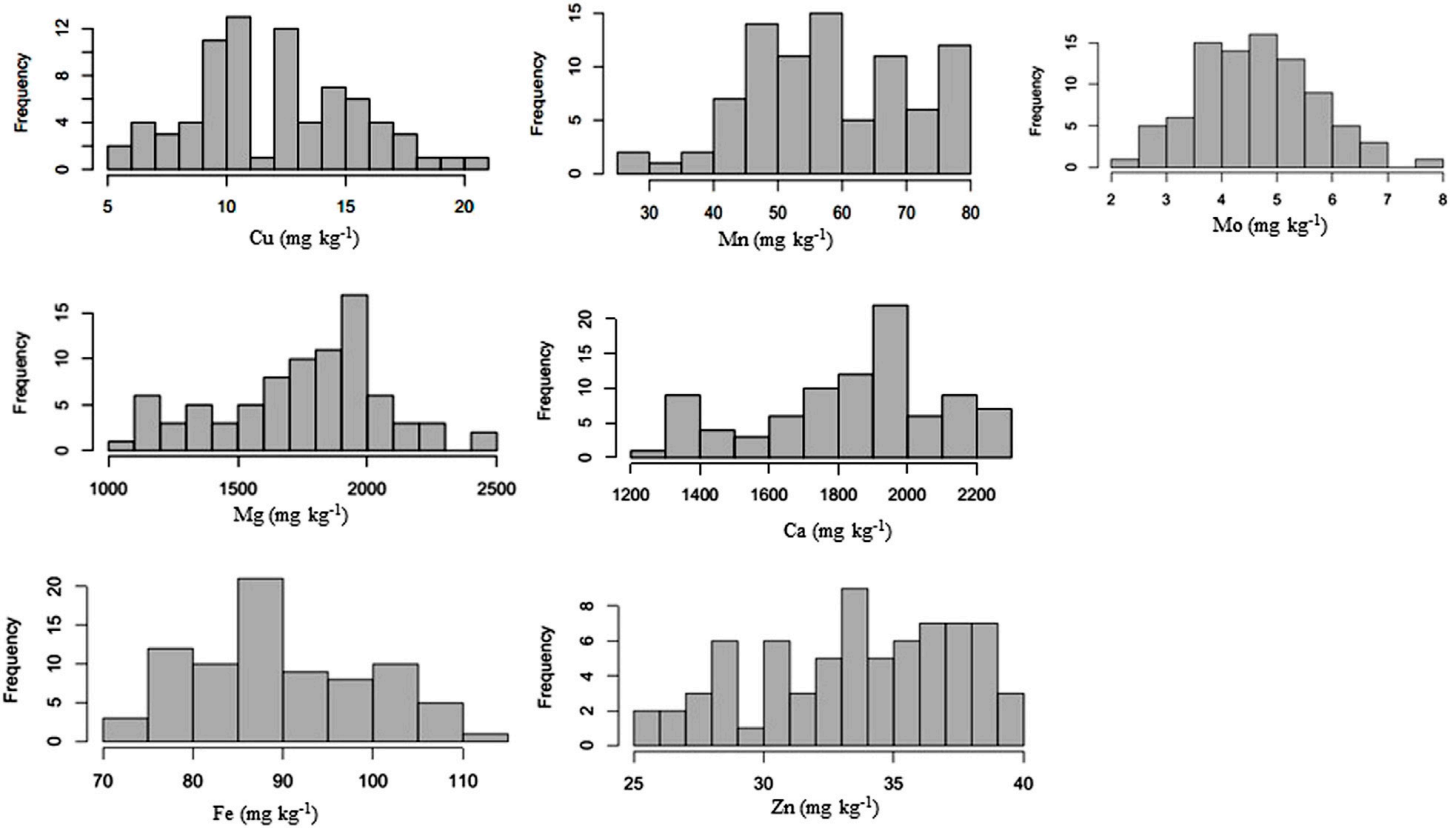
Frequency distribution of various micronutrients in common bean.

Micronutrient profiling of 96 genotypes was conducted to study the distribution and correlation among each other. Normal distribution was observed for Cu, Mn, Mg, Ca, Fe, Zn, and Mo (Figure 1; Supplementary Tables S2, S3). Seed micronutrient content showed continuous variation for Fe ranging from 67.35^1^–133.02 mg kg^−1^ (with average (av) of 94.21 mg kg^−1^), 21.16–49.77 mg kg^−1^ (av 35.98 mg kg^−1^) for Zn, 1,293.21–2,657.46 mg kg^−1^ (av 1893.56 mg kg^−1^) for Ca, 2.02–28.02 mg kg^−1^ (av 13.11 mg kg^−1^) for Cu, 1,057.55 mg kg^−1^ 208–2,492.26 mg kg^−1^ (av 1827.71 mg kg^−1^) for Mg, 22.34–93.36 mg kg^−1^ (av 58.26 mg kg^−1^) for Mn and 2.09–7.80 mg kg^−1^ (av 4.63 mg kg^−1^) for Mo (Table 1). Very low coefficients of variation (CVs) were observed for all these micronutrients.

**TABLE 1:** Range, highest, lowest genotype and Coefficient of Variance (CV) of different micronutrient content of common bean seeds.

S.No	Micronutrient	Range (mg kg^−1^)	Genotype		Average (mg kg^−1^±S.E)	CV
			Lowest	Highest		
1	Molybednum (Mo)	2.09–7.80	KD11	WB1680	4.63 ± 0.07	3.54
2	Zinc (Zn)	21.16–49.77	WB352	WB1190	35.98 ± 0.05	.30
3	Iron (Fe)	67.35–133.02	K13	WB1679	94.21 ± 0.04	.10
4	Calcium (Ca)	1,293.21–2,657.46	WB1643	KD7	1893.56 ± 0.03	.03
5	Magnesium (Mg)	1,057.55–2,492.26	WB371	K16	1827.71 ± 0.03	.004
6	Manganese (Mn)	22.34–93.36	R2	N15	58.26 ± 0.02	.27
7	Copper (Cu)	2.02–28.02	WB1136	UJ	13.11 ± 0.02	.07

### 3.2 Correlation Analysis

Pearson’s correlation analysis showed a highly significant positive correlation between Fe and Zn (r = .61**), Ca and Mg (r = .37**), Cu and Fe (r = .28**), and Cu and Zn (r = .26*) (Table 2). All the other micronutrients showed a non-significant correlation with each other. Correlation studies between the micronutrient contents of the seeds in the present study showed that Mn is the only micronutrient that correlates negatively with all other micronutrients such as Fe, Zn, Cu, Ca, Mg, and Mo. However, the Zn content was found positively correlated with Fe and Cu and negatively correlated with Ca, Mo, and Mg. Mo was found to correlate positively with Ca, Mg, Cu and negatively with Fe, Zn, and Mn. The Fe content was found to be positively correlated with Ca, Mg, and Cu, while it was negatively correlated with Mo and Mn.

**TABLE 2:** Correlation among seed micronutrient content in common bean.

Correlation							
	Mo	Zn	Fe	Ca	Mg	Mn	Cu
Mo	1						
Zn	−.104	1					
Fe	−.032	.608^a^	1				
Ca	.106	−.076	.086	1			
Mg	.052	−.029	.031	.373^a^	1		
Mn	−.010	−.140	−.125	−.028	−.010	1	
Cu	.121	.258^b^	.283^a^	.125	.150	−.183	1

a Correlation is significant at the .01 level.

b Correlation is significant at the .05 level.

### 3.3 Characterization and Distribution of SNPs in the Common Bean

**FIGURE 2:**
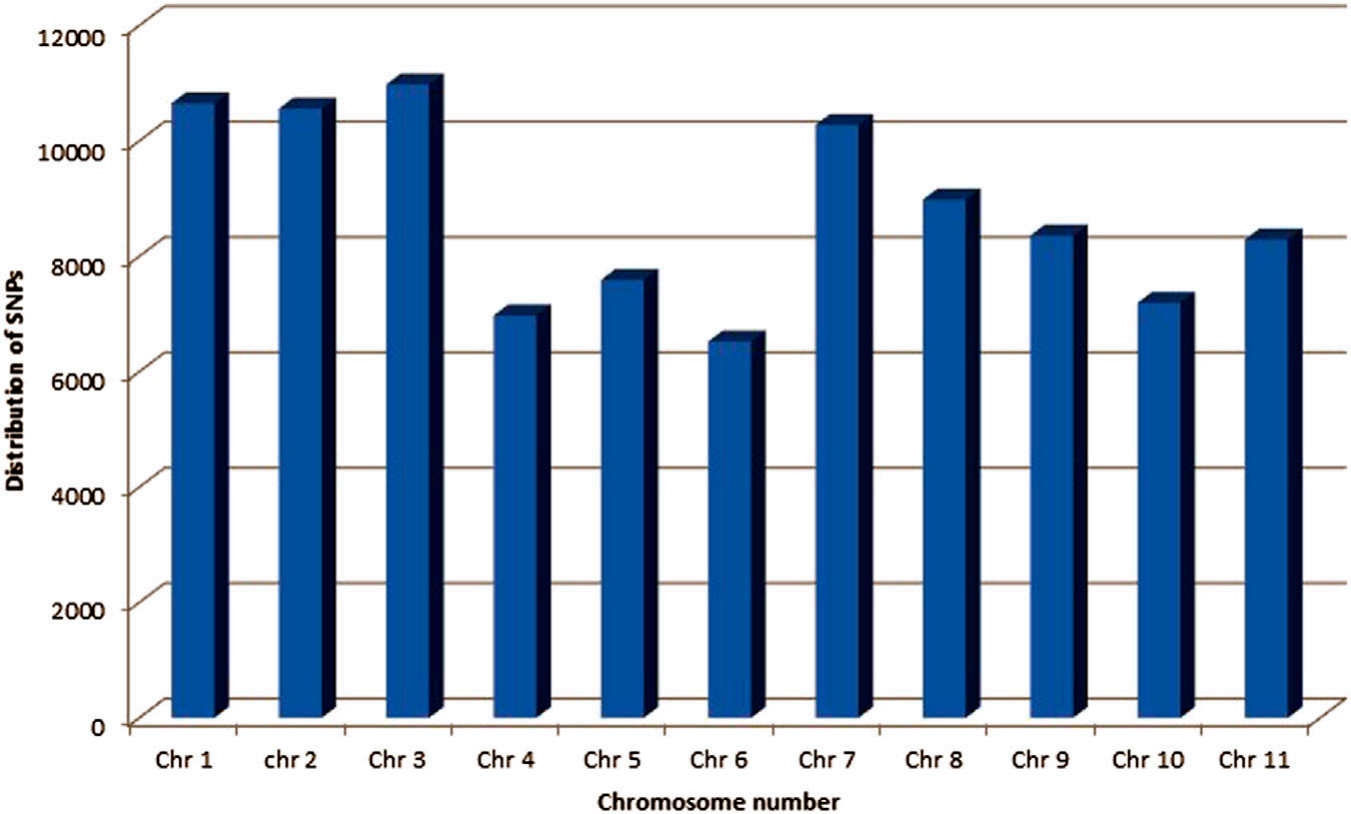
Frequency distribution of SNPs identified using GWAS on different chromosomes of common bean.

A total of 96,399 SNPs were found among a whole set of 96 diverse genotypes. The highest number of SNPs (10,978) were observed on chr. 3, whereas the lowest number of SNPs (6524) were observed on chr. 6 (Figure 2).

### 3.4 Genetic Diversity and Population Structure

**FIGURE 3:**
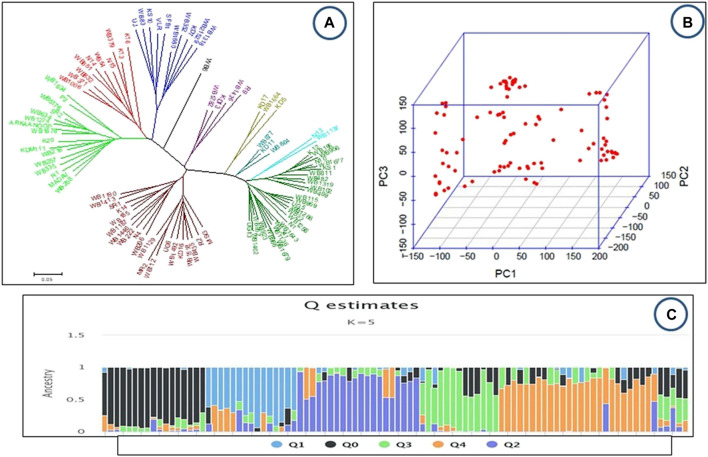
**(A–C)**: Genetic diversity and population structure of the studied common bean accessions **(A)** Phylogenetic trees constructed using the neighbor-joining method by Phiylip and MEGA 5 **(B)** PCA Scatter plots of the first two principal components (PCA analyses), each dot represents one accession **(C)**: Population structure K = 5, each accession is represented by a single vertical line and colors represent ancestries.

In the present study, all paired genetic distances between the 96 bean lines were determined from SNP-based genotypic data. A neighboring tree based on these genetic distances showed that the genotypes were divided into five main and five subgroups (Figure 3A). The Dendrogram revealed that of the minor groups, one included only one genotype, WB6; another group included only two genotypes (N13, WB1137); while another two groups were found to have only three genotypes each (KD17, WB1664, KD5 in one group and WB877, KD11, WB864 in the second group) and yet another group comprised of four genotypes (WB1282, KD13, R9, WB1436). The released lines like SR2 and ARKAANOOP were grouped in one cluster with other local lines, whereas VLR-125 and SFB1 clustered in another group.

Principal component analysis (PCA) also showed diversity among the common bean genotypes (Figure 3B). The released bean lines and local lines have been pooled with no clear separation of local and released lines (Figure 3B). In addition, population structure analysis provides a robust analysis for understanding the genotypic origins of a particular crop. The population structure was scored for K values ranging from 1 to 12 across the panel using high-quality SNPs. The peak of delta K was found to be the highest at K = 5 and thus groups the 96 genotypes of the common bean into 5 populations (Figure 3C). Furthermore, this was in accordance with the neighbor-joining tree with little deviation (Figure 3A).

### 3.5 Genome-Wide Association Analysis

**FIGURE 4:**
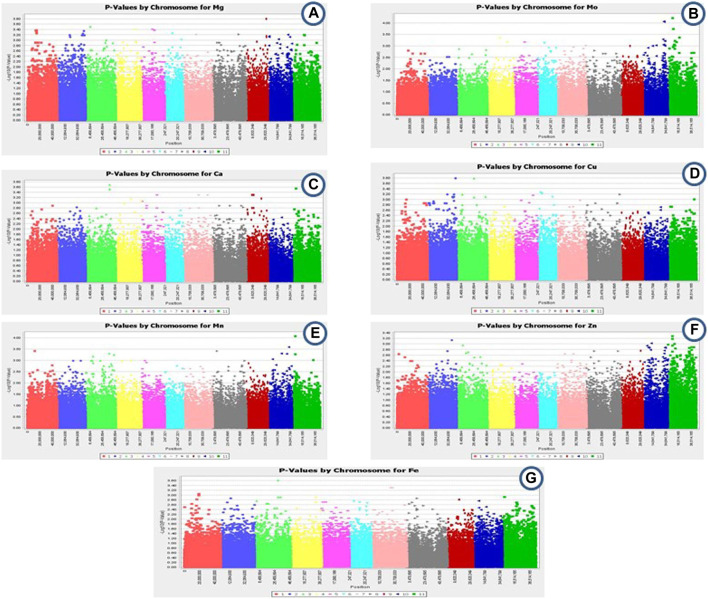
**(A–G)**: Summary of Genome-wide association result: Manhattan plots depicting association of 113 SNP markers with seed. **(A)** Mg, **(B)** Mo, **(C)** Ca, **(D)** Cu, **(E)** Mn, **(F)** Zn, **(G)**. Fe content in common bean.

GWAS was performed for common bean seed micronutrient contents (Fe, Zn, Cu, Ca, Mn, Mg, and Mo). Out of 96,399 SNPs, 113 SNPs were found to be significantly associated with different seed micronutrient contents with 13.50–21.74% phenotypic variance (Figures 4A–G; Table 3). A total of 32 SNPs across all chromosomes except chr. 8 were found significantly associated with seed Mg content. The highest number of SNPs (7) associated with seed Mg content was found on chr.2, while only one associated SNP was found on chr.1 and chr.7. One SNP on chr.9 positioned at 31752041 (*p*-value = 1.61E-04) contributed 18.42% phenotypic variation. For seed Mo content, 29 SNPs were found significantly associated that are positioned on chr.4, chr.5, chr.6, chr.7, chr.10, and chr.11 with the highest number of SNPs (13) on chr.10 and the lowest (1) on chr.6 and chr.7. One SNP on chr.11 contributed to 21.42% phenotypic variation. About 15 SNPs were found associated with seed Ca content on each of chr.3, chr.4, chr.5, chr. 7, chr. 9, and chr. 11 and seed Cu content on chr.2, chr.3, chr.5, chr.6, and chr.8. For seed Ca content, SNP contributing to the highest phenotypic variation (17.10%) was found on chr. 3 whereas, SNP associated to seed Cu content located on chr.2 and chr.3 contributed 21.74% and 21.71% phenotypic variation. Around 13 SNPs were found associated with seed Mn content on chr.1, chr.3, chr.8, chr.10 and chr.11 whereas, only 5 SNPs were found associated with seed Zn content on chr.2, chr.10, and chr.11 and 4 SNPs were associated to seed Fe content on chr.1, chr.3, and chr.7.

**TABLE 3:** Details of loci associated with accumulation of different elements.

Trait	Marker	Chr. No	Position	*p*-value	R_2_ (%)
Cu	Pv36231	2	43570203	1.63E-04	21.74
	Pv42447	3	26899343	1.64E-04	21.71
	Pv63237	6	4804173	5.52E-04	17.94
	Pv63769	6	7107934	6.00E-04	17.68
	Pv38848	3	8664946	6.52E-04	17.43 17.43
	Pv33235	2	30814539	6.52E-04	17.43
	Pv35821	2	41211297	6.52E-04	17.43
	Pv87597	8	52162199	6.52E-04	17.43
	Pv60686	5	29919387	7.02E-04	17.21
	Pv35247	2	38960183	8.03E-04	16.81
	Pv35810	2	41160625	8.03E-04	16.81
	Pv46872	3	44944379	8.03E-04	16.81
	Pv67269	6	23404896	8.08E-04	16.78
	Pv42479	3	27006768	8.36E-04	16.68
	Pv35442	2	39678800	8.46E-04	16.65
Mn	Pv18707	11	5168818	8.51E-05	20.40
	Pv16743	10	32828781	2.60E-04	17.38
	Pv80480	8	6963598	3.88E-04	16.33
	Pv02970	1	12045066	3.88E-04	16.33
	Pv45092	3	37444587	4.99E-04	15.67
	Pv14912	10	21149317	5.01E-04	15.66
	Pv14921	10	21209873	5.01E-04	15.66
	Pv16067	10	29484108	5.18E-04	15.57
	Pv18722	11	5199874	5.39E-04	15.47
	Pv46926	3	45208418	5.69E-04	15.33
	Pv39599	3	11581681	6.73E-04	14.89
	Pv12158	10	6845256	8.94E-04	14.17
	Pv24110	11	33811979	9.88E-04	13.91
Mg	Pv71125	9	31752041	1.61E-04	18.43
	Pv38472	3	7168425	3.16E-04	16.64
	Pv58364	5	17263012	3.92E-04	16.07
	Pv52919	4	30632382	3.96E-04	16.05
	Pv58963	5	20353152	4.32E-04	15.83
	Pv03198	1	13513718	4.33E-04	15.82
	Pv03216	1	13577463	4.33E-04	15.82
	Pv03452	1	14989418	4.33E-04	15.82
	Pv36071	2	42540272	4.57E-04	15.69
	Pv03386	1	14603432	5.19E-04	15.36
	Pv03456	1	14999140	5.19E-04	15.36
	Pv03454	1	14992813	5.58E-04	15.17
	Pv64956	6	12382652	5.66E-04	15.13
	Pv35797	2	41116882	5.76E-04	15.09
	Pv73343	7	20643908	6.13E-04	14.93
	Pv85139	8	41708004	6.13E-04	14.93
	Pv16441	10	31481349	6.29E-04	14.86
	Pv21626	11	19327905	6.39E-04	14.82
	Pv30574	2	20002969	6.51E-04	14.78
	Pv35396	2	39493707	6.64E-04	14.73
	Pv35747	2	40920106	6.64E-04	14.73
	Pv36214	2	43486371	6.64E-04	14.73
	Pv21789	11	20127048	6.64E-04	14.73
	Pv10966	10	1524933	7.14E-04	14.54
	Pv71157	9	31933306	7.14E-04	14.54
	Pv10985	10	1595959	7.66E-04	14.37
	Pv71179	9	32052922	7.66E-04	14.37
	Pv17142	10	34496599	7.66E-04	14.37
	Pv30756	2	20684206	7.70E-04	14.35
	Pv14063	10	16507514	9.28E-04	13.88
	Pv68046	6	26644872	9.73E-04	13.76
	Pv43881	3	32599558	9.98E-04	13.69
Ca	Pv45223	3	38019351	2.07E-04	17.10
	Pv18893	11	6191264	2.90E-04	16.25
	Pv45240	3	38092190	3.03E-04	16.13
	Pv45215	3	37988109	3.06E-04	16.11
	Pv65433	9	8733609	5.04E-04	14.87
	Pv66070	9	11355026	5.04E-04	14.87
	Pv66093	9	11451916	5.04E-04	14.87
	Pv66113	9	11504334	5.04E-04	14.87
	Pv59654	5	24452529	5.04E-04	14.87
	Pv73938	7	24798685	5.04E-04	14.87
	Pv77126	7	38956026	5.04E-04	14.87
	Pv69120	9	23846015	6.95E-04	14.08
	Pv51758	4	24279020	7.21E-04	13.99
	Pv54439	4	40294366	8.55E-04	13.58
	Pv54824	5	825380	8.80E-04	13.51
Fe	Pv43570	3	31289008	2.45E-04	18.82
	Pv74144	7	25770068	4.97E-04	16.81
	Pv74596	7	27864996	4.97E-04	16.81
	Pv04389	1	19220891	9.02E-04	15.15
Zn	Pv19080	11	7315158	5.31E-04	18.47
	Pv19085	11	7334690	6.62E-04	17.75
	Pv19088	11	7346014	6.62E-04	17.75
	Pv34942	2	37819199	7.36E-04	17.45
	Pv13503	10	12576659	9.54E-04	16.65
Mo	Pv19156	11	7595234	6.20E-05	21.42
	Pv16574	10	32099819	8.69E-05	20.49
	Pv16729	10	32768914	9.00E-05	20.40
	Pv19452	11	8815634	1.85E-04	18.43
	Pv21419	11	18293910	4.38E-04	16.13
	Pv51170	4	19580353	4.39E-04	16.12
	Pv16324	10	30756216	5.20E-04	15.68
	Pv20017	11	11345383	6.15E-04	15.24
	Pv16924	10	33461608	6.15E-04	15.24
	Pv16947	10	33552458	6.15E-04	15.24
	Pv16983	10	33671563	6.15E-04	15.24
	Pv58272	5	16746387	6.81E-04	14.97
	Pv58289	5	16870681	6.81E-04	14.97
	Pv53043	4	31371712	6.81E-04	14.97
	Pv20598	11	13856025	6.88E-04	14.95
	Pv64654	6	10916435	7.55E-04	14.71
	Pv19693	11	10155319	8.91E-04	14.28
	Pv19709	11	10212465	8.91E-04	14.28
	Pv16484	10	31648813	8.91E-04	14.28
	Pv16500	10	31724718	8.91E-04	14.28
	Pv16533	10	31876693	8.91E-04	14.28
	Pv16572	10	32090665	8.91E-04	14.28
	Pv14753	10	20089097	9.58E-04	14.10
	Pv19859	11	10700430	9.63E-04	14.08
	Pv19921	11	10908469	9.63E-04	14.08
	Pv78779	7	46163077	9.83E-04	14.03
	Pv17019	10	33837525	9.84E-04	14.03
	Pv20026	11	11415382	9.92E-04	14.00
	Pv16937	10	33511552	9.92E-04	14.00

### 3.6 LD Plot and Haplotype Blocks

**FIGURE 5:**
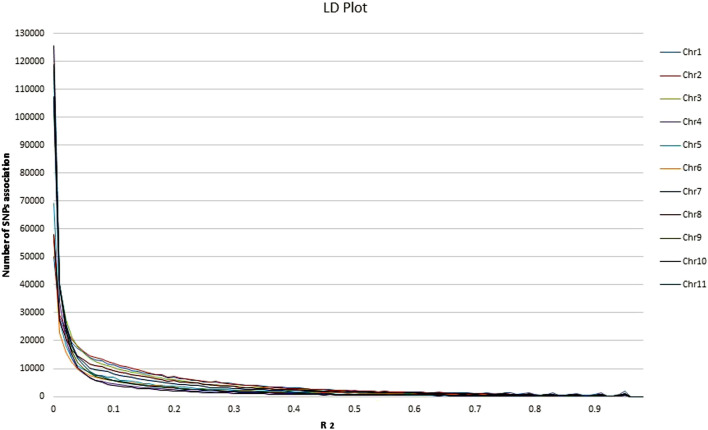
Analysis of linkage disequilibrium (LD) plot across the 11 common bean chromosomes.

**FIGURE 6:**
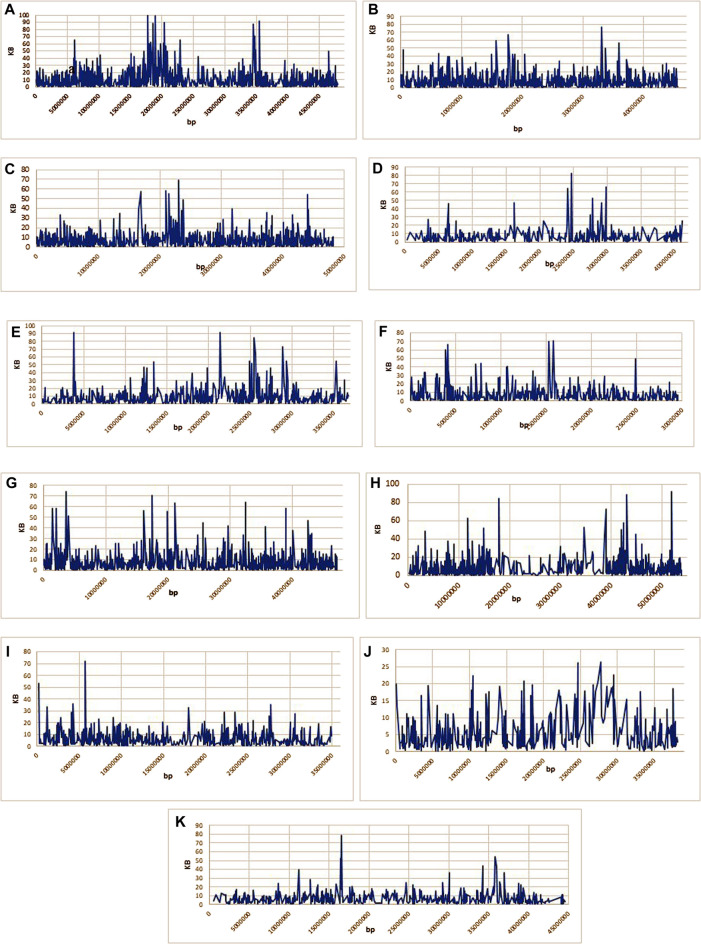
**(A–K)**: Representing the haploblocks of 11 common bean chromosomes.

Overall LD measured as R^2^ was correlated in all chromosomes (Figure 5; Supplementary Table S4). The maximum number of SNPs (125,580) correlated with other chromosomes were found on chr.10, whereas, a minimum number of SNPs (50,003) were found on chr.1 at R^2^ = .01. With the increase in R^2^ value, decreasing pattern in the number of associated SNPs across the 11 chromosomes was observed. At R^2^ = .93 the lowest number of SNPs correlation was found in all the chromosomes. The lowest (33) and highest (232) were found on chr. 6 and 1, respectively. Moreover, a total of 1879 SNPs on chr.1 were found to correlate with other chromosomes at R^2^ = .96, whereas 421 SNPs were found on chr.10. No SNPs were found associated across 11 chromosomes at R^2^ = .97–.99. Through haplotype analysis, a set of 7107 haploblocks representative of the 11 chromosomes, ranging from 1244 (chr. 1) to 503 (chr. 4) were identified. A total of 22,090 SNPs were distributed in these blocks, with an average of ∼3 SNPs per block. Chr. 1 (17) and chr. 4 and 10 (7 each) had the highest and lowest number of SNPs within their haploblocks, respectively (Figures 6A–K; Supplementary Table S5).

### 3.7 Candidate Gene Analysis

A total of 840 genes were identified in the .1 Mb flanking region of significant SNPs related to different traits; however, five SNPs were not co-localized with any gene in the .1 Mb flanking region (Supplementary Table S6). Out of these 840 genes, 16 transporter genes were identified **(** Table 4), some being metal transporters. We have also identified genes for 24 metal-binding proteins (Table 4 **)** like zinc finger, calcium, and iron-binding proteins.

**TABLE 4:** Identified gene related to transporter/metal transporter and metal ion binding proteins.

QTL name	Gene ID	Chr name	Start	End	Strand	Description
QTL_Zn_2_37819199	Phvul.002G210700	Chr02	37776912	37782162	−1	ABC-2 TYPE TRANSPORTER
QTL_Zn_2_37819199	Phvul.002G210500	Chr02	37746541	37750458	1	ABC TRANSPORTER
QTL_Zn_2_37819199	Phvul.002G210600	Chr02	37769769	37772725	−1	ABC TRANSPORTER
QTL_Mn_1_12045066	Phvul.001G079200	Chr01	12009949	12013454	−1	CALCIUM-BINDING TRANSPORTER-LIKE PROTEIN
QTL_Mg_1_13513718	Phvul.001G087300	Chr01	13547691	13551468	1	ZINC FINGER PROTEIN CONSTANS-LIKE 14-RELATED
QTL_Mg_1_13577463	Phvul.001G087600	Chr01	13639243	13640935	−1	ZINC FINGER PROTEIN CONSTANS-LIKE 14-RELATED
QTL_Zn_2_37819199	Phvul.002G211500	Chr02	37863083	37865132	−1	NITRATE, FROMATE, IRON DEHYDROGENASE
QTL_Mg_2_39493707	Phvul.002G223300	Chr02	39466477	39468933	1	OXIDOREDUCTASE, 2OG-FE II OXYGENASE FAMILY PROTEIN
QTL_Cu_2_39678800	Phvul.002G224601	Chr02	39628554	39636892	1	ZINC FINGER FYVE DOMAIN CONTAINING PROTEIN//SUBFAMILY NOT NAMED
QTL_Cu_2_39678800	Phvul.002G226100	Chr02	39760216	39762026	−1	SNF2, HELICASE AND ZINC-FINGER DOMAIN-CONTAINING PROTEIN-RELATED
QTL_Mg_2_43486371/QTL_Cu_2_43570203	Phvul.002G264200	Chr02	43553358	43564776	−1	DOF DOMAIN, ZINC FINGER (ZF-DOF)
QTL_Mg_2_43486371	Phvul.002G262500	Chr02	43427768	43428934	−1	COPPER TRANSPORT PROTEIN ATOX1-RELATED
QTL_Mn_3_37444587	Phvul.003G157800	Chr03	37403436	37405196	−1	3.6.3.41 - HEME-TRANSPORTING ATPASE (1 OF 1)
QTL_Ca_3_38092190	Phvul.003G162600	Chr03	38180169	38182817	1	ZINC FINGER CCCH DOMAIN-CONTAINING PROTEIN 39
QTL_Ca_3_38092190	Phvul.003G162500	Chr03	38160568	38162888	1	ZINC FINGER PROTEIN
QTL_Ca_3_38092190	Phvul.003G162400	Chr03	38151881	38153482	-1	ZINC FINGER PROTEIN
QTL_Cu_3_44944379	Phvul.003G221000	Chr03	44958664	44959509	-1	C2H2-TYPE ZINC FINGER
QTL_Mn_3_45208418	Phvul.003G223100	Chr03	45277144	45282157	1	OXIDOREDUCTASE, 2OG-FE II OXYGENASE FAMILY PROTEIN
QTL_Ca_5_825380	Phvul.005G008400	Chr05	739318	742827	−1	FOLATE-BIOPTERIN TRANSPORTER 8, CHLOROPLASTIC-RELATED
QTL_Cu_6_7107934	Phvul.006G015300	Chr06	7024910	7028768	1	CALCIUM-DEPENDENT PROTEIN KINASE 17-RELATED
QTL_Mg_6_26644872	Phvul.006G164200	Chr06	26717778	26719484	−1	PREDICTED TRANSPORTER (MAJOR FACILITATOR SUPERFAMILY)
QTL_Mg_6_26644872	Phvul.006G163300	Chr06	26634344	26644212	−1	ZINC FINGER CCCH DOMAIN-CONTAINING PROTEIN 34-RELATED
QTL_Mg_7_20643908	Phvul.007G111200	Chr07	20693634	20695218	-1	CALCIUM BINDING PROTEIN
QTL_Ca_7_24798685	Phvul.007G149300	Chr07	24734652	2,4741700	-1	MEMBRANE MAGNESIUM TRANSPORTER (MMGT)
QTL_Fe_7_25770068	Phvul.007G152700	Chr07	2,5821756	25822875	1	CALCIUM/CALMODULIN-DEPENDENT PROTEIN KINASE
QTL_Fe_7_27864996	Phvul.007G165166	Chr07	27868704	27875618	−1	MITOCHONDRIAL METAL TRANSPORTER 1-RELATED
QTL_Ca_7_38956026	Phvul.007G267600	Chr07	38871259	38872086	1	DOF DOMAIN, ZINC FINGER (ZF-DOF
QTL_Mn_8_6963598	Phvul.008G074100	Chr08	7022615	7031313	−1	C2 CALCIUM/LIPID-BINDING ENDONUCLEASE/EXONUCLEASE/PHOSPHATASE-RELATED
QTL_Cu_8_52162199	Phvul.008G185000	Chr08	52129836	52132078	1	CALCIUM-ACTIVATED CHLORIDE CHANNEL REGULATOR
QTL_Ca_9_8733609	Phvul.009G043800	Chr09	8788738	8789490	−1	C2H2-TYPE ZINC FINGER (ZF-C2H2_6)
QTL_Ca_9_8733609	Phvul.009G042600	Chr09	8633736	8637094	1	PROBABLE ZINC-RIBBON DOMAIN (ZINC_RIBBON_12)
QTL_Ca_9_8733609	Phvul.009G043700	Chr09	8779518	8780270	−1	C2H2-TYPE ZINC FINGER (ZF-C2H2_6)
QTL_Mo_10_31876693	Phvul.010G071100	Chr10	31841020	31842480	−1	C2H2-TYPE ZINC FINGER (ZF-C2H2_6)
QTL_Mo_10_31876693	Phvul.010G071300	Chr10	31949277	31951593	1	C2H2-LIKE ZINC FINGER PROTEIN-RELATED
QTL_Mn_11_5168818/QTL_Mn_11_5199874	Phvul.011G058100	Chr11	5183094	5185016	−1	AN1-TYPE ZINC FINGER PROTEIN
QTL_Mn_11_5168818/QTL_Mn_11_5199874	Phvul.011G058500	Chr11	5228604	5232591	1	ZINC TRANSPORTER, ZIP FAMILY (TC.ZIP, ZUPT, ZRT3, ZIP2)
QTL_Ca_11_6191264	Phvul.011G068700	Chr11	6106515	6108448	−1	COPPER TRANSPORT PROTEIN ATOX1-RELATED
QTL_Mo_11_7595234	Phvul.011G081250	Chr11	7526302	7531699	−1	CALCIUM-ACTIVATED CHLORIDE CHANNEL REGULATOR
QTL_Mo_11_10155319/QTL_Mo_11_10212465	Phvul.011G098800	Chr11	10254133	10256987	−1	ALUMINUM-ACTIVATED MALATE TRANSPORTER 1-RELATED
QTL_Mo_11_10700430	Phvul.011G100400	Chr11	10792608	10796881	1	CALCIUM-DEPENDENT PHOSPHOTRIESTERASE SUPERFAMILY PROTEIN-RELATED
QTL_Mo_11_11345383/QTL_Mo_11_11415382	Phvul.011G102500	Chr11	11388457	11393199	−1	MULTI-COPPER OXIDASE

## 4 Discussion

### 4.1 Micronutrients Variation and Correlation Among Micronutrients

Micronutrients play an indispensable role in the growth and development of eukaryotic organisms. A deficiency in these essential micro-and macronutrients leads to abnormal growth in living systems. Humans get most of their micronutrients from plant and animal sources. Therefore, the biofortification of important food crops is necessary nowadays. In the present study, an initiative was taken to investigate different micronutrient concentrations in the germplasm of common beans. The micronutrient content of the seeds has been varied widely in common bean seeds (Supplementary Table S2). Previous studies have shown that mineral variation has been observed in almost all major legumes, including the common bean. The different mineral content in beans has been studied in different parts of the world including India (O Abidemi et al., 2012; Martinez Meyer et al., 2013; Zaccardelli et al., 2013; Kumar and Chopra, 2014; Mahajan R et al., 2015; Erdogmus et al., 2020). Earlier reports also suggested that seed mineral content showed huge variation in common bean germplasm. Seed mineral concentrations such as Mg, Ca, Fe, Zn, and Cu of 60 common bean genotypes collected from the Western Himalayas varied from 1,220.5 to 2,737.5 ppm, 300–5,350 ppm, 80.5–180.6 ppm for Fe, 14.64–104.08 ppm, and .9–13.4 ppm, respectively (Jan et al., 2021). The average seed Ca concentration was recorded as 1.37 and 1.41 g kg^−1^, whereas, the average Fe seed content was recorded as 79.57 and 85.95 mg kg^−1^ from common bean seeds obtained through pedigree and single seed descent methods respectively (Ribeiro et al., 2014). Also, 88.14 mg kg^−1^of Fe, 49.24 mg kg^−1^of Zn, .25 g 100 g^−1^ of Mg, 11.30 mg kg^−1^of Cu, and 22.71 mg kg^−1^of Mn was found in common bean genotypes from Universidade Federal de Lavras (UFLA) (Silva et al., 2010) and 74.6 ppm Fe, 39.9 ppm Zn content in Ugandan common bean germplasm (Mukamuhirwa et al., 2012). The difference in micronutrient content in bean genotypes from different parts of the world may be due to the different number of samples taken for evaluation, environmental conditions such as climate and soil composition, and agricultural techniques. This diversity in the germplasm can help us identify potential candidate lines that can be used in the development of Multi-Parent Advanced Generation Intercross (MAGIC) or bi-parental mapping populations, the breeding of micronutrient-rich, high-yielding varieties of beans, can be used for investigating different levels of gene expression for different nutrients in common beans. The higher micronutrient bean lines could be used for biofortification programs.

Based on the Pearson’s correlation analysis our study reveals a significant positive correlation between Fe and Zn; Ca and Mg; Cu, Fe, and Zn. In previous studies, a similar correlation pattern between Zn and Fe and other minerals was observed in bean genotypes (Beebe et al., 2000; House et al., 2002; Gelin et al., 2007; Pfeiffer and McClafferty, 2007). Correlation studies have been conducted on other agronomical traits in the common bean (Nadeem et al., 2020). The negative correlation between the trait suggests that these traits are interdependent. In the present study, Mn was found negatively correlated with all other micronutrients, which means with the increase in Mn concentration there will be a decrease in the concentration of other micronutrients in common bean seeds. In addition, a positive correlation indicated that increasing the concentration of one micronutrient would positively affect the concentration of other micronutrients.

### 4.2 GWAS for Micronutrients

Genetic diversity is an important parameter for studying variability in any crop and identifying superior alleles controlling qualitative and quantitative traits through association mapping (Nachimuthu et al., 2015). Molecular markers such as SSRs and SNPs have an important role in studying genetic diversity in most crops (Nachimuthu et al., 2015; Zargar et al., 2016). Insights into the genomic diversity and population structure of common bean germplasm can expedite the genetic gains in common bean-breeding programs (Blair et al., 2012; Blair et al., 2013). The diversity based on the dissimilarity coefficient divided selected germplasm into five main groups and subgroups. The results of the clustering showed that the local germplasm of the common bean of Jammu & Kashmir (J&K) is very diverse and could be used as advanced lines for the genetic enhancement of the common bean. Further, intermixing of released and local lines collected from different regions of J&K indicated that the selected germplasm is diverse. Some of the previously conducted diversity studies on local landraces of the common beans collected from the J&K region have also divided the germplasm into different groups (Zargar et al., 2016; Mahajan et al., 2017a; Mahajan et al., 2017b). In order to have knowledge about the sub-populations in a particular crop structure analysis was performed.

Based on the sharp peak for the delta-K value and the results of the PCA, 96 common bean genotypes were classified into five major groups. A similar pattern of population structure K = 5 was also found in common bean germplasm from Jammu and Kashmir (Mahajan et al., 2017a; Mahajan et al., 2017b). However, earlier studies also classified the common bean germplasm into K = 2 (Gupta et al., 2020; Nkhata et al., 2020; Mir et al., 2021); K = 3 (Blair et al., 2012; Blair et al., 2013; Dennis et al., 2014; Nemli et al., 2014). The difference in cluster and structure analysis could be due to different algorithms used by the two approaches. The cluster analysis is based on evolutionary dissimilarity, while the population structure is based on a Bayesian algorithm. The deviations in the results can be attributed to the different germplasm, the different marker system, and the different geographical locations.

For the identification of genes associated with different traits in a large population, GWAS offers much higher mapping resolutions (Mamo et al., 2014; Norton et al., 2014). To the best of our knowledge, there are only a few association studies on different traits in common bean germplasm collected from the Himalayan region (Mahajan et al., 2017b; Choudhary et al., 2018; Gupta et al., 2020; Mir et al., 2021). However, in earlier studies, genic and genomic SSRs were used for GWAS. The present study is the first report on the association of SNPs related to seed micronutrient content in common bean germplasm from the Himalayan region. In the present study, it was found that SNPs associated with most of the seed micronutrients (Mg, Mn, Fe, Ca, Cu) were found on chr.3 and chr.11 (Mg, Mn, Mo, Ca, Zn) whereas, chr.8 have SNPs associated with Cu, Mg and Mn and chr.9 have SNPs associated with only Mg and Ca. Earlier studies revealed that QTLs linked with Fe content were found on chr. 2, 5, 6, 7, 9, and 10, whereas Zn content was found on chr.1, 2, 3, 5, 7, 8, and 10 (Blair et al., 2011; Mahajan et al., 2017a). In a recent study, a single QTL on chr. 9 and chr. 8 was found to be associated with seed Ca content and seed Mg content, respectively, whereas two QTLs on chr. 6 were associated with seed Zn content (Gunjača et al., 2021). In our study, we found that all of the 11 chromosomes have SNPs associated with more than one seed micronutrient content, which indicates the preciseness of using high throughput genotypic data in the present study.

### 4.3 LD Plot and Haploblock in Common Bean Germplasm

Knowledge about the LD properties in domesticated crops is important as it underlies all types of genetic mapping and may be used in the fine mapping of genes associated with complex traits in crop plants. It is said that in-depth LD in a crop is important for having more SNP-based associations to predict the average number of markers required for GWAS (Nordborg and Tavaré, 2002). Identification of QTLs inassociation mapping is based on Linkage Disequilibrium measurement. The population structure and genetic relatedness between the genotypes can lead to false-positive LD analysis. LD pattern in germplasm is affected by reducing genetic diversity by various factors like the type of selection, population admixture (Contreras-Soto et al., 2017). Our study is in accordance with the previous common bean LD estimation studies (Erdogmus et al., 2020; Gunjača et al., 2021). Hence, we can conclude that as the number of SNPs increases, there will be more R^2^ and the higher the likelihood of association of markers with traits of interest. This also indicated that a significant association would be possible with LD block having a higher number of SNPs compared to those windows having lower SNPs. The present study suggests that GBS is an advanced approach to analyzing genetic diversity and population structure in the common bean. The haplotype-based analysis showed that more haplocks were found in the centromeric region than in the telomeric region. In Chr.10 (26,295 kb) higher haplotypes per kb were found than in the rest of the chromosomes, which suggests that the LD decay in Chr.10 is stronger than in other chromosomes.

## 5 Candidate Genes

GWAS is often used for comparative genome analysis and helps explicitly in dissection as well as in understanding the complex quantitative feature analysis. The GWAS helps identify significant SNPs associated with a trait that is not the function of the region of interest, and sometimes these SNPs are present in non-coding or non-regulatory regions of chromosomes (Bararyenya et al., 2020). Thus, it is important to identify the candidate genes in the vicinity of the significant SNPs, and in our study, the significant SNP regions were examined for the identification of putative protein-coding genes using the *P. vulgaris* genome. The genes that are present in the .1 Mb flanking region of significant SNPs are given in Supplementary Table S6. Many previous studies also reported some markers linked with traits but no genes in the genomic region of markers (Bararyenya et al., 2020). The number of genes in SNPs revealed that chromosome 2, 6, 9, and 11 has more number of genes in 0.1 MB flanking region of SNPs has a high density of genes or the hot spot of QTLs.

## 6 Conclusion

Micronutrient deficiency is the leading cause of human health deterioration worldwide. An animal or plant-based diet alone cannot provide humans with excessive amounts of essential micronutrients. Biofortification of food crops is therefore important in order to provide humans with essential micronutrients. Modern breeding approaches such as QTL mapping and association mapping are important to identify QTLs that are related to micronutrient levels in plants. In the present study, a significant variation in the micronutrient content of the seeds in the germplasm of common beans was found. The present data on genetic loci, particularly the key SNPs associated with seven elements, will be helpful in identifying candidate genes, understanding molecular mechanisms, and developing molecular markers for breeding applications. We firmly believe that the results of the current studies will help accelerate bean biofortification efforts to overcome nutritional deficiencies.

## Data Availability

The data sets presented in this study can be found in online repositories. The names of the repository/repositories and accession number(s) can be found in the article/Supplementary Material.
