# Supplementary material for: Identification of QTLs/ Candidate Genes for Seed Mineral Contents in Common Bean (Phaseolus vulgaris L.) Through Genotyping-by-Sequencing
Source: Front Genet. 2022 Mar 14;13:750814. doi: 10.3389/fgene.2022.750814 (PMC8982075; doi:10.3389/fgene.2022.750814)
Supplement: Supplementary file 3 [file DataSheet2.docx]

Supplementary Table 1: List of common bean genotypes along with their source of procurement

| S.No. | Genotype code | Source of Procurement | S.No. | Genotype code | Source of Procurement |
| --- | --- | --- | --- | --- | --- |
| 1 | **MSG** | Shangardund, Sopore | 49 | **WB877** | Nadihal Baramulla |
| 2 | **WB258** | Dachan Kishtwar | 50 | **P2** | Poonch |
| 3 | **WB1006** | Yunsu Sopore | 51 | **WB195** | WaduraSopore |
| 4 | **KD17** | Kashmir | 52 | **WB6** | Unknown |
| 5 | **WB1680** | Markoot Gurez | 53 | **WB1286** | Pulwama |
| 6 | **N13** | Nishat | 54 | **WB22** | Unknown |
| 7 | **WB206** | Wadura Sopore | 55 | **WB1274** | Dangerpora |
| 8 | **R2** | Rajouri | 56 | **SR2** | Released variety |
| 9 | **K12** | Bandipora | 57 | **KD11** | Kashmir |
| 10 | **SFB1** | Selection from HOS-1 (IIPR Kanpur) | 58 | **WB5176** | Uri |
| 11 | **WB352** | Unknown | 59 | **WB1679** | Baghtore Gurez |
| 12 | **WB1282** | Shopian | 60 | **WB634** | Kralpora |
| 13 | **WB435** | Unknown | 61 | **K20** | Qazigund |
| 14 | **WB1664** | Anantnag | 62 | **N15** | Nishat |
| 15 | **VLR** | Almora | 63 | **WB1492** | Gurez |
| 16 | **KD7** | Kashmir | 64 | **KD5** | Kashmir |
| 17 | **N4** | Nishat | 65 | **K13** | Baramulla |
| 18 | **WB1643** | Unknown | 66 | **WB482** | Unknown |
| 19 | **WB341** | Shopian | 67 | **WB966** | Unknown |
| 20 | **WB185** | Wadura | 68 | **K16** | Bandipora |
| 21 | **K14** | Bandipora | 69 | **WB102** | Anantnag |
| 22 | **WB115** | Shopian | 70 | **WB222** | Shopian |
| 23 | **WB969** | Wadura | 71 | **WB371** | Unknown |
| 24 | **KS10** | Shopian | 72 | **WB1402** | Doda |
| 25 | **KD16** | Kashmir | 73 | **WB923** | TujarSopore |
| 26 | **N10** | Nishat | 74 | **R9** | Rajouri |
| 27 | **WB21529** | Kupwara | 75 | **WB489** | Unknown |
| 28 | **WB216** | Wadura | 76 | **UG13** | Ugada |
| 29 | **KDM111** | - | 77 | **WB83** | HygamSopore |
| 30 | **WB651** | Lawaypora Bandipora | 78 | **N1** | Nishat |
| 31 | **KD13** | Kashmir | 79 | **WB1190** | Logripora |
| 32 | **WB1436** | Lolab | 80 | **WB1187** | Kupwara |
| 33 | **WB1678** | BoglinderTulain | 81 | **WB1446** | Unknown |
| 34 | **WB54** | WaduraSopore | 82 | **UJ** | Baramulla |
| 35 | **WB112** | Ananatnag | 83 | **WB956** | WaduraSopore |
| 36 | **UG5** | Ugada | 84 | **WB832** | ChakdaraBandopora |
| 37 | **N14** | Nishat | 85 | **WB257** | SaidporaSopore |
| 38 | **WB379** | Unknown | 86 | **WB952** | Sopore |
| 39 | **WB1518** | Wadura | 87 | **WB1136** | Shopian |
| 40 | **MR2** | Marmat | 88 | **WB335** | Unknown |
| 41 | **WB1129** | Pulwama | 89 | **WB1634** | Wadura |
| 42 | **KS1** | Shopian | 90 | **WB864** | NaidkhaiSopore |
| 43 | **MADAV** | Anantnag | 91 | **WB811** | WarporaSopore |
| 44 | **WB1318** | Poonch | 92 | **WB1256** | DangerporaSopore |
| 45 | **WB1413** | Shopian | 93 | **WB1137** | Shopian |
| 46 | **UG6** | Ugada | 94 | **SR1** | Canadian Red x Local Red |
| 47 | **ARKAANOOP** | Released variety IIHR Bangalore | 95 | **R1** | Rajouri |
| 48 | **WB1677** | Keegam Kupwara | 96 | **WB1319** | Sutharan Budgam |

Supplementary Table 2:Seed micronutrient content (mean value ± S.E) in different common beans genotypes

| MEAN±S.E (mg kg^-1^) | | | | | | | | |
| --- | --- | --- | --- | --- | --- | --- | --- | --- |
| S.NO. | Genotype | Mo | Zn | Fe | Ca | Mg | Mn | Cu |
| 1 | **MSG** | 3.5±0.05 | 30.36±0.03 | 89.63±0.05 | 1956.25±0.03 | 1896.29±0.02 | 76.28±0.01 | 14.62±0.01 |
| 2 | **WB258** | 3.76±0.02 | 28.54±0.03 | 86.82±0.02 | 1968.27±0.01 | 1958.15±0.02 | 44.14±0.01 | 11.10±0.05 |
| 3 | **WB1006** | 2.82±0.03 | 31.36±0.03 | 78.82±0.02 | 2245.36±0.02 | 1925.23±0.02 | 75.27±0.01 | 10.04±0.04 |
| 4 | **KD17** | 3.82±0.00 | 37.35±0.04 | 98.57±0.03 | 1808.09±0.04 | 1103.91±0.02 | 22.59±0.01 | 8.24±0.02 |
| 5 | **WB1680** | 7.8±0.05 | 38.68±0.05 | 110.71±0.02 | 1955.24±0.02 | 1750.62±0.01 | 54.35±0.11 | 12.24±0.02 |
| 6 | **N13** | 5.71±0.06 | 33.3±0.01 | 88.52±0.04 | 1345.24±0.01 | 1965.22±0.02 | 44.46±0.01 | 15.09±0.05 |
| 7 | **WB206** | 3.81±0.01 | 28.34±0.03 | 90.94±0.02 | 1786.90±0.00 | 1689.60±0.20 | 58.54±0.01 | 13.03±0.02 |
| 8 | **R2** | 6.22±0.01 | 30.71±0.03 | 81.92±0.02 | 1789.36±0.02 | 2289.37±0.02 | 22.33±0.01 | 16.81±0.00 |
| 9 | **K12** | 4.51±0.02 | 25.54±0.04 | 79.57±0.07 | 1856.18±0.01 | 1785.66±0.02 | 88.54±0.01 | 11.02±0.01 |
| 10 | **SFB1** | 4.16±0.03 | 38.55±0.07 | 99.94±0.02 | 2253.94±0.03 | 2308.24±0.02 | 32.72±0.02 | 24.15±0.01 |
| 11 | **WB352** | 5.55±0.01 | 21.16±0.06 | 78.17±0.03 | 1896.24±0.02 | 1768.32±0.01 | 66.55±0.01 | 10.03±0.02 |
| 12 | **WB1282** | 4.42±0.03 | 35.22±0.06 | 104.82±0.03 | 1954.47±0.02 | 1586.25±0.02 | 56.43±0.02 | 7.82±0.01 |
| 13 | **WB435** | 3.730.00± | 32.35±0.14 | 102.66±0.27 | 1879.66±0.02 | 1745.26±0.03 | 85.45±0.01 | 16.00±0.00 |
| 14 | **WB1664** | 3.75±0.01 | 26.69±0.07 | 91.56±0.05 | 1954.6±0.01 | 1786.12±0.01 | 75.45±0.00 | 10.02±0.01 |
| 15 | **VLR** | 2.71±0.01 | 48.75±0.07 | 116.6±0.09 | 1464.45±0.02 | 1153.37±0.02 | 68.42±0.01 | 14.57±0.01 |
| 16 | **KD7** | 5.01±0.02 | 40.73±0.06 | 126.25±0.05 | 2657.46±0.02 | 2345.54±0.02 | 43.57±0.00 | 16.81±0.01 |
| 17 | **N4** | 5.32±0.05 | 36.57±0.05 | 85.82±0.03 | 1723.78±0.01 | 1540.89±0.02 | 49.14±0.02 | 11.03±0.01 |
| 18 | **WB1643** | 4.42±0.0 | 37.66±0.06 | 102.72±0.03 | 1293.21±0.02 | 2335.96±0.01 | 61.87±0.01 | 16.03±0.03 |
| 19 | **WB341** | 4.57±0.03 | 31.69±0.05 | 97.25±0.04 | 1992.61±0.03 | 2354.22±0.01 | 55.33±0.02 | 5.00±0.00 |
| 20 | **WB185** | 7.69±0.00 | 32.30±0.01 | 87.37±0.02 | 2478.84±0.02 | 2420.10±0.00 | 36.33±0.02 | 23.54±0.01 |
| 21 | **K14** | 4.01±0.00 | 32.64±0.03 | 85.48±0.03 | 1301.21±0.02 | 1165.25±0.02 | 58.22±0.01 | 12.01±0.01 |
| 22 | **WB115** | 4.74±0.02 | 33.65±0.05 | 85.77±0.03 | 1747.50±0.02 | 2465.14±0.01 | 57.35±0.01 | 17.22±0.01 |
| 23 | **WB969** | 5.02±0.01 | 45.42±0.04 | 89.26±0.03 | 1644.09±0.05 | 1338.68±0.12 | 82.68±0.01 | 9.41±0.01 |
| 24 | **KS10** | 4.49±0.02 | 33.92±0.02 | 87.10±0.05 | 2010.24±0.04 | 1156.53±0.02 | 65.74±0.02 | 5.52±0.01 |
| 25 | **KD16** | 2.73±0.04 | 48.94±0.02 | 128.62±0.05 | 1378.02±0.01 | 1692.20±0.02 | 50.05±0.02 | 7.01±0.00 |
| 26 | **N10** | 4.85±0.02 | 41.48±0.03 | 94.91±0.02 | 1596.5±0.1 | 1863.43±0.02 | 45.63±0.02 | 14.61±0.00 |
| 27 | **WB21529** | 4.62±0.02 | 44.76±0.02 | 87.66±0.04 | 2001.38±0.03 | 1879.23±0.01 | 66.21±0.01 | 9.01±0.00 |
| 28 | **WB216** | 2.43±0.02 | 41.44±0.12 | 93.91±0.02 | 1341.72±0.02 | 2026.83±0.02 | 90.23±0.01 | 8.61±0.00 |
| 29 | **KDM111** | 4.57±0.01 | 33.75±0.03 | 74.89±0.02 | 2256.23±0.02 | 1923.56±0.06 | 74.14±0.01 | 7±0.00 |
| 30 | **WB651** | 5.20±0.02 | 37.86±0.02 | 78.75±0.02 | 1874.55±0.03 | 1758.35±0.02 | 66.25±0.01 | 4.01±0.01 |
| 31 | **KD13** | 5.47±0.01 | 27.92±0.03 | 74.77±0.02 | 1862.54±0.02 | 1826.55±0.27 | 63.36±0.00 | 14.11±0.00 |
| 32 | **WB1436** | 5.10±0.01 | 30.15±0.04 | 82.06±0.03 | 2245.27±0.02 | 2145.27±0.02 | 78.23±0.01 | 7.01±0.01 |
| 33 | **WB1678** | 3.54±0.02 | 28.76±0.02 | 77.93±0.02 | 1535.6±0.01 | 2200.87±0.04 | 47.06±0.02 | 12.46±0.04 |
| 34 | **WB54** | 2.66±0.03 | 35.85±0.03 | 88.04±0.02 | 1358.26±0.03 | 1654.43±0.02 | 47.45±0.02 | 12.81±0.00 |
| 35 | **WB112** | 4.26±0.04 | 43.57±0.04 | 104.92±0.02 | 1374.21±0.02 | 1290.69±0.01 | 54.25±0.01 | 12.81±0.01 |
| 36 | **UG5** | 6.56±0.02 | 38.56±0.05 | 85.56±0.03 | 1847.42±0.01 | 2432.22±0.01 | 58.77±0.00 | 17.24±0.03 |
| 37 | **N14** | 7.61±0.01 | 36.69±0.02 | 82.24±0.04 | 2189.77±0.01 | 2001.23±0.01 | 45.24±0.00 | 13.01±0.01 |
| 38 | **WB379** | 7.52±0.01 | 36.88±0.04 | 110.89±0.03 | 1959.25±0.03 | 1857.52±0.01 | 48.53±0.01 | 10.02±0.01 |
| 39 | **WB1518** | 6.80±0.05 | 29.23±0.05 | 103.09±0.05 | 2145.26±0.02 | 2014.23±0.01 | 77.84±001 | 16.02±0.01 |
| 40 | **MR2** | 5.70±0.01 | 45.84±0.03 | 113.19±0.04 | 2551.25±0.02 | 2374.54±0.02 | 45.54±0.01 | 23.53±0.01 |
| 41 | **WB1129** | 3.69±0.02 | 28.37±0.02 | 80.55±0.05 | 2124.32±0.11 | 1989.24±0.01 | 53.24±0.00 | 21.01±0.01 |
| 42 | **KS1** | 5.63±0.02 | 46.99±0.00 | 107.90±0.02 | 1955.57±0.01 | 2211.21±0.01 | 92.15±0.02 | 24.03±0.02 |
| 43 | **MADAV** | 4.82±0.01 | 28.07±0.03 | 79.81±0.01 | 1879.53±0.15 | 1657.13±0.02 | 78.21±0.01 | 11.02±0.01 |
| 44 | **WB1318** | 3.8±0.01 | 27.57±0.02 | 85.47±0.02 | 1999.33±0.02 | 1985.44±0.00 | 68.74±0.02 | 4.03±0.01 |
| 45 | **WB1413** | 3.99±0.06 | 33.45±0.09 | 109.64±0.06 | 1567.17±0.03 | 1368.27±0.00 | 64.14±0.02 | 11.02±0.01 |
| 46 | **UG6** | 5.35±0.07 | 27.08±0.02 | 103.16±0.03 | 2237.85±0.01 | 1338.68±0.01 | 61.5±0.1 | 19.82±0.01 |
| 47 | **ARKAANOOP** | 4.16±0.08 | 36.74±0.02 | 104.49±0.03 | 1714.60±0.08 | 1654.45±0.02 | 45.52±0.01 | 23.22±0.01 |
| 48 | **WB1677** | 5.7±0.11 | 32.16±0.02 | 76.97±0.04 | 1929.34±0.02 | 2441.32±0.01 | 52.23±0.01 | 12.8±0.01 |
| 49 | **WB877** | 3.67±0.10 | 41.93±0.03 | 110.08±0.04 | 2457.85±0.02 | 1376.43±0.02 | 56.17±0.01 | 14.24±0.04 |
| 50 | **P2** | 4.52±0.17 | 39.06±0.05 | 82.23±0.06 | 2076.71±0.04 | 2144.56±0.03 | 44.69±0.01 | 20.62±0.02 |
| 51 | **WB195** | 3.73±0 | 36.17±0.01 | 88.64±0.04 | 2120.06±0.03 | 1977.33±0.00 | 51.42±0.01 | 9.22±0.01 |
| 52 | **WB6** | 5.66±0.17 | 34.21±0.01 | 94.56±0.03 | 1345.21±0.01 | 1220.73±0.01 | 51.96±0.02 | 10.83±0.01 |
| 53 | **WB1286** | 5.03±0.17 | 38.63±0.02 | 68.9±0.03 | 1755.54±0.02 | 1663.16±0.02 | 57.60±0.00 | 11.03±0.03 |
| 54 | **WB22** | 4.35±0.01 | 34.63±0.04 | 100.14±0.04 | 1750.81±0.02 | 1666.52±0.01 | 52.24±0.02 | 10.02±0.01 |
| 55 | **WB1274** | 6.06±0.14 | 32.07±0.03 | 85.57±0.03 | 2210.21±0.06 | 2008.15±0.01 | 70.26±0.00 | 13.023±0.01 |
| 56 | **SR2** | 4.07±0.11 | 45.65±0.03 | 94.83±0.03 | 1745.59±0.01 | 1410.26±0.01 | 29.05±0.01 | 9.73±0.02 |
| 57 | **KD11** | 2.09±0.05 | 40.41±0.06 | 108.76±0.03 | 2445.33±0.02 | 2395.12±0.00 | 29.44±0.02 | 13.11±0.06 |
| 58 | **WB5176** | 5.41±0.12 | 35.91±0.02 | 88.57±0.02 | 1989.46±0.01 | 1884.79±0.01 | 76.29±0.02 | 17.03±0.02 |
| 59 | **WB1679** | 4.33±0.09 | 41.87±0.03 | 133.02±0.02 | 1931.59±0.06 | 2308.84±0.02 | 61.85±0.01 | 19.01±0.00 |
| 60 | **WB634** | 4.62±0.10 | 36.44±0.05 | 97.33±0.02 | 1981.28±0.02 | 1875.82±0.01 | 48.47±0.01 | 16.01±0.00 |
| 61 | **K20** | 4.71±0.08 | 36.37±0.07 | 101.54±0.04 | 1832.44±0.02 | 2031.82±0.01 | 68.46±0.01 | 20.05±0.01 |
| 62 | **N15** | 6.50±0.03 | 33.89±0.02 | 92.92±0.03 | 2135.66±0.02 | 1971.53±0.02 | 93.35±0.01 | 9.83±0.02 |
| 63 | **WB1492** | 4.99±0.11 | 45.04±0.03 | 128.8±0.02 | 2010.13±0.02 | 1780.55±0.01 | 55.23±0.01 | 12.43±0.03 |
| 64 | **KD5** | 3.51±0.10 | 35.08±0.04 | 98.92±0.02 | 1711.77±0.01 | 2452.12±0.01 | 51.77±0.00 | 10.43±0.02 |
| 65 | **K13** | 3.50±0.06 | 28.26±0.02 | 67.35±0.04 | 1827.55±0.02 | 1263.65±0.01 | 24.26±0.01 | 8.22±0.01 |
| 66 | **WB482** | 5.19±0.05 | 32.63±0.05 | 77.54±0.02 | 1987.27±0.02 | 1789.53±0.01 | 59.25±0.00 | 5.04±0.02 |
| 67 | **WB966** | 4.26±0.08 | 37.86±0.03 | 124.24±0.03 | 2275.54±0.02 | 1958.42±0.01 | 41.43±0.01 | 15.46±0.04 |
| 68 | **K16** | 2.66±0.08 | 37.87±0.01 | 103.28±0.02 | 2547.55±0.03 | 2492.25±0.03 | 37.86±0.02 | 13.25±0.04 |
| 69 | **WB102** | 5.53±0.17 | 38.56±0.06 | 109.57±0.03 | 1978.52±0.01 | 1920.27±0.01 | 48.17±0.01 | 8.00±0.00 |
| 70 | **WB222** | 4.28±0.11 | 42.1±0.04 | 96.64±0.03 | 1656.35±0.02 | 1186.53±0.02 | 46.93±0.02 | 17.22±0.02 |
| 71 | **WB371** | 5.34±0.13 | 30.53±0.19 | 90.173±0.05 | 1848.44±0.02 | 1057.55±0.01 | 53.62±0.02 | 9.41±0.00 |
| 72 | **WB1402** | 6.38±0.08 | 41.76±0.03 | 108.77±0.02 | 1409.21±0.02 | 1585.24±0.01 | 24.26±0.00 | 12.42±0.01 |
| 73 | **WB923** | 3.34±0.13 | 33.31±0.15 | 84.65±0.03 | 1986.24±0.02 | 1874.58±0.03 | 76.26±0.01 | 16.02±0.01 |
| 74 | **R9** | 4.52±0.15 | 35.90±0.01 | 87.14±0.04 | 1697.91±0.02 | 1197.65±0.01 | 46.23±0.06 | 12.22±0.02 |
| 75 | **WB489** | 5.36±0.08 | 34.27±0.07 | 71.87±0.03 | 1695.46±0.01 | 1589.23±0.02 | 45.85±0.02 | 24.02±0.02 |
| 76 | **UG13** | 4.54±0.15 | 33.14±0.04 | 80.50±0.01 | 1340.53±0.01 | 1943.48±0.01 | 77.84±0.02 | 14.22±0.01 |
| 77 | **WB83** | 3.273±0.14 | 39.15±0.13 | 75.83±0.04 | 1985.60±0.01 | 1931.45±0.01 | 42.34±0.02 | 28±0.00 |
| 78 | **N1** | 2.74±0.07 | 43.18±0.04 | 100.27±0.04 | 1874.26±0.02 | 1496.62±0.01 | 45.07±0.01 | 10.72±0.01 |
| 79 | **WB1190** | 3.78±0.03 | 49.77±0.00 | 122.26±0.04 | 2145.33±0.01 | 2005.44±0.01 | 76.27±0.02 | 14.03±0.02 |
| 80 | **WB1187** | 3.49±0.01 | 37.94±0.02 | 75.72±0.02 | 2004.25±0.02 | 1874.22±0.01 | 66.26±0.02 | 11.02±0.01 |
| 81 | **WB1446** | 7.48±0.18 | 37.11±0.01 | 110.50±0.03 | 2496.24±0.01 | 1356.93±0.01 | 55.23±0.02 | 23.62±0.01 |
| 82 | **UJ** | 4.80±0.07 | 40.94±0.02 | 120.16±0.06 | 1656.17±0.01 | 1061.34±0.02 | 71.25±0.02 | 28.02±0.01 |
| 83 | **WB956** | 4.8±0 | 38.84±0.02 | 98.25±0.04 | 1987.43±0.02 | 1835.17±0.02 | 56.45±0.01 | 13.81±0.01 |
| 84 | **WB832** | 4.13±0.09 | 40.56±0.04 | 88.85±0.03 | 1957.13±0.02 | 1911.14±0.01 | 75.24±0.02 | 6.01±0.01 |
| 85 | **WB257** | 6.64±0.09 | 38.90±0.01 | 111.60±0.04 | 1476.23±0.02 | 2147.52±0.01 | 56.98±0.03 | 10.62±0.01 |
| 86 | **WB952** | 2.56±0.04 | 30.39±0.05 | 87.52±0.02 | 1982.14±0.02 | 1869.45±0.1 | 75.26±0.02 | 5.01±0.01 |
| 87 | **WB1136** | 3.33±0.11 | 25.84±0.03 | 79.5±0.03 | 2144.74±0.02 | 1987.23±0.01 | 68.29±0.01 | 2.02±0.01 |
| 88 | **WB335** | 5.61±0.05 | 34.13±0.03 | 87.31±0.04 | 1328.22±0.01 | 1756.06±0.03 | 69.36±0.01 | 10.17±0.01 |
| 89 | **WB1634** | 5.26±0.14 | 34.68±0.11 | 89.77±0.03 | 1402.21±0.01 | 1453.62±0.01 | 50.78±0.01 | 10.6±0.01 |
| 90 | **WB864** | 5.89±0.01 | 33.66±0.05 | 77.55±0.04 | 2122.34±0.02 | 1988.15±0.02 | 69.32±0.02 | 5.01±0.01 |
| 91 | **WB811** | 3.71±0.05 | 26.71±0.02 | 80.81±0.01 | 1998.55±0.02 | 1935.45±0.00 | 56.14±0.02 | 8.02±0.01 |
| 92 | **WB1256** | 4.25±0.15 | 30.56±0.02 | 83.33±0.04 | 1988.25±0.02 | 1785.24±0.01 | 56.86±0.02 | 10.02±0.01 |
| 93 | **WB1137** | 5.97±0.04 | 35.64±0.03 | 90.54±0.05 | 2100.45±0.01 | 1999.84±0.02 | 74.14±0.01 | 7.00±0.00 |
| 94 | **SR1** | 2.18±0.04 | 48.92±0.02 | 130.1±0.04 | 2000.44±0.01 | 2448.85±0.01 | 44.17±0.01 | 23.53±0.01 |
| 95 | **R1** | 3.44±0.06 | 40.26±0.03 | 82.27±0.02 | 1668.95±0.02 | 1518.25±0.02 | 73.54±0.01 | 14.01±0.01 |
| 96 | **WB1319** | 4.71±0.05 | 39.63±0.07 | 99.46±0.04 | 1874.25±0.02 | 1657.34±0.02 | 73.21±0.01 | 17.01±0.01 |
| AVG. |  | 4.63±0.07 | 35.98±0.05 | 94.21±0.04 | 1893.56±0.03 | 1827.71±0.03 | 58.26±0.02 | 13.11±0.02 |
| **Max** |  | **7.80** | **49.77** | **133.03** | **2657.46** | **2492.26** | **93.36** | **28.02** |
| **Min** |  | **2.09** | **21.16** | **67.36** | **1293.21** | **1057.55** | **22.34** | **2.02** |
| **C.D** |  | **0.18** | **0.12** | **0.10** | **0.07** | **0.09** | **0.03** | **0.05** |
| **C.V** |  | **3.54** | **0.30** | **0.10** | **0.03** | **0.004** | **0.27** | **0.07** |

**Supplementary Table 3: ANNOVA table for analyzed elements**

| **Source of variation** | **df** | Mo | | Zn | | Fe | | Ca | | Mn | | Mg | | Cu | |
| --- | --- | --- | --- | --- | --- | --- | --- | --- | --- | --- | --- | --- | --- | --- | --- |
|  |  | MSS | F Ratio | MSS | F Ratio | MSS | F Ratio | MSS | F Ratio | MSS | F Ratio | MSS | F Ratio | MSS | F Ratio |
| Genotype | 95 | 4.826 | 225.18*** | 105.861 | 11470*** | 669.54 | 94880*** | 289742.5 | 78860000*** | 757.424 | 518900*** | 407489.7 | 73160000*** | 92.53 | 69960*** |
| Error | 190 | 0.021 |  | 0.009 |  | 0.007 |  | 0.004 |  | 0.001 |  | 0.006 |  | 0.001 |  |

Supplementary Table 4: LD plot analysis of all 11 chromosomes

| Chr1 | Chr2 | Chr3 | Chr4 | Chr5 | Chr6 | Chr7 | Chr8 | Chr9 | Chr10 | Chr11 | R2BinMax |
| --- | --- | --- | --- | --- | --- | --- | --- | --- | --- | --- | --- |
| 50003 | 56034 | 102681 | 105836 | 69137 | 57744 | 107554 | 57919 | 118972 | 125580 | 117258 | 0.01 |
| 27963 | 29321 | 39478 | 33956 | 27256 | 22870 | 40141 | 27181 | 39355 | 39805 | 40197 | 0.02 |
| 22825 | 24089 | 27542 | 20129 | 18220 | 15706 | 25951 | 20728 | 23900 | 23218 | 25122 | 0.03 |
| 19129 | 20485 | 21364 | 13628 | 13729 | 12138 | 18675 | 16691 | 16380 | 15168 | 18007 | 0.04 |
| 17171 | 18122 | 17702 | 9953 | 11158 | 9838 | 14370 | 14693 | 12213 | 10588 | 13750 | 0.05 |
| 15710 | 16151 | 15463 | 8168 | 9517 | 8554 | 12193 | 13114 | 9917 | 8248 | 10953 | 0.06 |
| 14060 | 14698 | 13571 | 6815 | 8550 | 7548 | 10250 | 11555 | 8240 | 6610 | 9007 | 0.07 |
| 13180 | 14045 | 12522 | 5850 | 7466 | 6812 | 9677 | 10943 | 7234 | 5777 | 7938 | 0.08 |
| 12649 | 13253 | 11667 | 5387 | 7405 | 6455 | 9350 | 10611 | 6731 | 5045 | 7204 | 0.09 |
| 11759 | 12393 | 11055 | 4875 | 6937 | 6155 | 8911 | 9742 | 6170 | 4335 | 6321 | 0.1 |
| 11412 | 11896 | 10418 | 4507 | 6853 | 5659 | 8215 | 9304 | 5705 | 3974 | 5812 | 0.11 |
| 10552 | 10889 | 9667 | 4229 | 5886 | 5271 | 7598 | 8679 | 5103 | 3756 | 5368 | 0.12 |
| 10237 | 10704 | 9406 | 3957 | 5754 | 5088 | 7251 | 8519 | 4827 | 3518 | 5228 | 0.13 |
| 9404 | 10187 | 8810 | 3700 | 5313 | 4884 | 6834 | 7846 | 4520 | 3253 | 4692 | 0.14 |
| 9002 | 9524 | 8318 | 3485 | 5141 | 4486 | 6384 | 7588 | 4306 | 2833 | 4219 | 0.15 |
| 8315 | 8869 | 7718 | 3232 | 4681 | 4182 | 6108 | 7202 | 3981 | 2698 | 3987 | 0.16 |
| 8027 | 8489 | 7451 | 3026 | 4557 | 4051 | 5815 | 6787 | 3793 | 2655 | 3676 | 0.17 |
| 7795 | 7939 | 6884 | 2859 | 4084 | 3873 | 5531 | 6201 | 3594 | 2423 | 3477 | 0.18 |
| 7468 | 7787 | 6587 | 2733 | 3893 | 3710 | 5152 | 6048 | 3370 | 2293 | 3335 | 0.19 |
| 6767 | 6926 | 6032 | 2530 | 3640 | 3305 | 4883 | 5582 | 3165 | 2127 | 2958 | 0.2 |
| 7062 | 7240 | 5948 | 2449 | 3648 | 3256 | 4705 | 5633 | 3239 | 1956 | 3080 | 0.21 |
| 6412 | 6626 | 5600 | 2230 | 3330 | 2912 | 4280 | 5210 | 2873 | 1822 | 2801 | 0.22 |
| 6360 | 6462 | 5421 | 2274 | 3324 | 2925 | 4352 | 5105 | 2790 | 1803 | 2648 | 0.23 |
| 5676 | 5933 | 5030 | 1955 | 3061 | 2511 | 3875 | 4794 | 2561 | 1671 | 2449 | 0.24 |
| 5713 | 5810 | 4742 | 1974 | 2881 | 2499 | 3902 | 4736 | 2601 | 1531 | 2519 | 0.25 |
| 5284 | 5554 | 4484 | 1822 | 2722 | 2403 | 3537 | 4397 | 2451 | 1454 | 2267 | 0.26 |
| 5046 | 5096 | 4114 | 1682 | 2562 | 2107 | 3156 | 4137 | 2203 | 1377 | 2192 | 0.27 |
| 5026 | 5435 | 4350 | 1773 | 2602 | 2280 | 3166 | 4105 | 2215 | 1364 | 2106 | 0.28 |
| 4881 | 4818 | 4073 | 1598 | 2395 | 2070 | 3082 | 3653 | 1967 | 1253 | 1993 | 0.29 |
| 4859 | 4629 | 3896 | 1526 | 2416 | 2035 | 3060 | 3639 | 2002 | 1244 | 2022 | 0.3 |
| 4434 | 4278 | 3669 | 1528 | 2358 | 1918 | 2835 | 3598 | 1807 | 1059 | 1895 | 0.31 |
| 4408 | 4306 | 3585 | 1418 | 2173 | 1800 | 2750 | 3166 | 1662 | 1099 | 1764 | 0.32 |
| 3861 | 4023 | 3198 | 1379 | 2141 | 1800 | 2647 | 3063 | 1697 | 1057 | 1689 | 0.33 |
| 3933 | 4008 | 3377 | 1316 | 2098 | 1835 | 2570 | 3028 | 1614 | 963 | 1588 | 0.34 |
| 3984 | 3657 | 2975 | 1226 | 1990 | 1755 | 2507 | 2907 | 1529 | 951 | 1527 | 0.35 |
| 3586 | 3312 | 2974 | 1222 | 1931 | 1644 | 2404 | 2838 | 1509 | 912 | 1395 | 0.36 |
| 3532 | 3499 | 2840 | 1224 | 1979 | 1630 | 2339 | 2868 | 1398 | 890 | 1429 | 0.37 |
| 3268 | 2984 | 2647 | 1052 | 1799 | 1618 | 2224 | 2511 | 1329 | 836 | 1364 | 0.38 |
| 3353 | 3052 | 2742 | 1111 | 1806 | 1534 | 2133 | 2544 | 1199 | 826 | 1424 | 0.39 |
| 2992 | 2933 | 2323 | 1013 | 1706 | 1472 | 2054 | 2400 | 1221 | 766 | 1244 | 0.4 |
| 2977 | 2899 | 2584 | 992 | 1728 | 1447 | 2160 | 2363 | 1272 | 713 | 1242 | 0.41 |
| 3061 | 2765 | 2363 | 942 | 1622 | 1443 | 2110 | 2271 | 1168 | 706 | 1228 | 0.42 |
| 2822 | 2683 | 2397 | 927 | 1683 | 1467 | 1995 | 2144 | 1190 | 680 | 1213 | 0.43 |
| 2632 | 2426 | 2074 | 861 | 1561 | 1406 | 1918 | 2095 | 1035 | 599 | 1091 | 0.44 |
| 2540 | 2393 | 2148 | 825 | 1536 | 1336 | 1871 | 1943 | 1051 | 609 | 1114 | 0.45 |
| 2482 | 2377 | 2019 | 823 | 1521 | 1281 | 1852 | 1766 | 1015 | 559 | 968 | 0.46 |
| 2481 | 2359 | 1992 | 794 | 1511 | 1284 | 1780 | 1889 | 1000 | 604 | 1008 | 0.47 |
| 2265 | 2098 | 1872 | 743 | 1396 | 1201 | 1713 | 1773 | 885 | 526 | 838 | 0.48 |
| 2198 | 2252 | 1915 | 763 | 1361 | 1247 | 1758 | 1688 | 877 | 524 | 874 | 0.49 |
| 2042 | 2154 | 1735 | 674 | 1240 | 1103 | 1532 | 1612 | 825 | 474 | 761 | 0.5 |
| 2143 | 2012 | 1749 | 726 | 1328 | 1218 | 1598 | 1642 | 867 | 479 | 759 | 0.51 |
| 1910 | 1921 | 1586 | 716 | 1227 | 1150 | 1515 | 1643 | 836 | 467 | 684 | 0.52 |
| 2048 | 1869 | 1478 | 625 | 1230 | 1092 | 1542 | 1515 | 874 | 445 | 688 | 0.53 |
| 1835 | 1894 | 1422 | 587 | 1226 | 1064 | 1539 | 1435 | 822 | 419 | 674 | 0.54 |
| 1670 | 1746 | 1323 | 560 | 1059 | 1043 | 1397 | 1237 | 758 | 392 | 676 | 0.55 |
| 1919 | 1840 | 1348 | 560 | 1121 | 989 | 1441 | 1541 | 747 | 418 | 667 | 0.56 |
| 1606 | 1630 | 1216 | 548 | 1058 | 1060 | 1371 | 1368 | 754 | 398 | 557 | 0.57 |
| 1671 | 1508 | 1331 | 540 | 1051 | 979 | 1451 | 1345 | 747 | 386 | 600 | 0.58 |
| 1674 | 1488 | 1126 | 462 | 1022 | 911 | 1333 | 1245 | 766 | 372 | 601 | 0.59 |
| 1609 | 1462 | 1109 | 471 | 971 | 852 | 1310 | 1192 | 685 | 350 | 597 | 0.6 |
| 1748 | 1395 | 1205 | 479 | 1008 | 1038 | 1361 | 1214 | 733 | 362 | 585 | 0.61 |
| 1524 | 1485 | 1033 | 481 | 970 | 922 | 1388 | 1185 | 638 | 310 | 566 | 0.62 |
| 1394 | 1310 | 980 | 420 | 991 | 893 | 1178 | 1140 | 667 | 384 | 530 | 0.63 |
| 1348 | 1199 | 983 | 442 | 915 | 893 | 1284 | 1083 | 663 | 314 | 525 | 0.64 |
| 1484 | 1201 | 962 | 429 | 981 | 860 | 1183 | 1004 | 669 | 289 | 482 | 0.65 |
| 1403 | 1227 | 996 | 414 | 857 | 813 | 1100 | 964 | 634 | 309 | 515 | 0.66 |
| 1248 | 1151 | 895 | 427 | 894 | 843 | 997 | 952 | 560 | 273 | 443 | 0.67 |
| 1273 | 1124 | 980 | 352 | 938 | 807 | 1160 | 1044 | 556 | 271 | 470 | 0.68 |
| 1260 | 1079 | 853 | 367 | 813 | 798 | 1025 | 883 | 621 | 282 | 575 | 0.69 |
| 1274 | 1011 | 841 | 375 | 712 | 698 | 980 | 910 | 588 | 273 | 477 | 0.7 |
| 997 | 891 | 757 | 383 | 810 | 785 | 981 | 881 | 544 | 228 | 444 | 0.71 |
| 1078 | 1080 | 732 | 332 | 820 | 753 | 993 | 908 | 516 | 257 | 401 | 0.72 |
| 1263 | 1033 | 749 | 326 | 739 | 655 | 915 | 934 | 529 | 257 | 541 | 0.73 |
| 1131 | 774 | 763 | 292 | 662 | 587 | 869 | 655 | 519 | 209 | 398 | 0.74 |
| 856 | 825 | 647 | 241 | 742 | 682 | 911 | 659 | 516 | 205 | 345 | 0.75 |
| 1241 | 1029 | 678 | 319 | 802 | 749 | 988 | 836 | 503 | 227 | 435 | 0.76 |
| 1203 | 847 | 610 | 246 | 594 | 552 | 692 | 697 | 432 | 226 | 477 | 0.77 |
| 684 | 510 | 514 | 213 | 443 | 445 | 625 | 384 | 402 | 205 | 347 | 0.78 |
| 904 | 597 | 580 | 246 | 720 | 640 | 871 | 566 | 485 | 213 | 309 | 0.79 |
| 1378 | 807 | 759 | 281 | 729 | 574 | 770 | 928 | 475 | 200 | 400 | 0.8 |
| 891 | 503 | 539 | 164 | 451 | 357 | 509 | 441 | 291 | 186 | 328 | 0.81 |
| 542 | 317 | 373 | 161 | 360 | 360 | 581 | 285 | 319 | 156 | 214 | 0.82 |
| 852 | 603 | 611 | 267 | 731 | 651 | 825 | 572 | 447 | 199 | 328 | 0.83 |
| 1188 | 724 | 603 | 244 | 686 | 527 | 596 | 735 | 393 | 215 | 381 | 0.84 |
| 481 | 395 | 280 | 157 | 296 | 205 | 310 | 289 | 220 | 167 | 231 | 0.85 |
| 454 | 389 | 291 | 138 | 298 | 221 | 390 | 255 | 285 | 118 | 192 | 0.86 |
| 659 | 457 | 509 | 223 | 604 | 550 | 716 | 470 | 448 | 187 | 268 | 0.87 |
| 1117 | 614 | 579 | 251 | 632 | 572 | 569 | 653 | 381 | 211 | 408 | 0.88 |
| 338 | 212 | 210 | 107 | 138 | 83 | 112 | 149 | 103 | 90 | 145 | 0.89 |
| 400 | 275 | 212 | 110 | 240 | 154 | 268 | 240 | 180 | 95 | 150 | 0.9 |
| 616 | 354 | 420 | 252 | 435 | 400 | 554 | 359 | 397 | 212 | 296 | 0.91 |
| 1382 | 743 | 619 | 319 | 629 | 513 | 651 | 687 | 389 | 238 | 445 | 0.92 |
| 232 | 119 | 133 | 43 | 41 | 33 | 69 | 66 | 80 | 52 | 83 | 0.93 |
| 298 | 159 | 139 | 77 | 99 | 92 | 140 | 102 | 131 | 93 | 135 | 0.94 |
| 631 | 321 | 321 | 148 | 295 | 231 | 378 | 313 | 291 | 173 | 290 | 0.95 |
| 1879 | 866 | 725 | 505 | 975 | 756 | 998 | 948 | 659 | 421 | 722 | 0.96 |
| 0 | 0 | 0 | 0 | 0 | 0 | 0 | 0 | 0 | 0 | 0 | 0.97 |
| 0 | 0 | 0 | 0 | 0 | 0 | 0 | 0 | 0 | 0 | 0 | 0.98 |
| 0 | 0 | 0 | 0 | 0 | 0 | 0 | 0 | 0 | 0 | 0 | 0.99 |
